# Bioclimatic niches are conserved and unrelated to pollination syndromes in Antillean Gesneriaceae

**DOI:** 10.1098/rsos.170293

**Published:** 2017-11-01

**Authors:** Hermine Alexandre, Julie Faure, Steven Ginzbarg, John Clark, Simon Joly

**Affiliations:** 1 Institut de Recherche en Biologie Végétale, Université de Montréal, 4101 Sherbrooke East, Montreal, Quebec, Canada H1X2B2; 2 Department of Biological Sciences, The University of Alabama, Tuscaloosa, AL 35487-0344, USA; 3 Science Department, The Lawrenceville School, Lawrenceville, NJ 08648-1699, USA; 4 Montreal Botanical Garden, 4101 Rue Sherbrooke E, Montreal, Quebec, Canada H1X 2B2

**Keywords:** bioclimatic niche, biotic interaction, niche conservatism, island, Ornstein-Uhlenbeck models, phylogenetic comparative analyses, environmental space

## Abstract

The study of the evolution of abiotic niches can be informative regarding the speciation drivers in a given group. Yet, two factors that could potentially affect niche evolution have seldom been addressed concomitantly, which are biotic interactions and geographical isolation. In this study, we used as a model group the Antillean plant genera *Gesneria* and *Rhytidophyllum* (Gesneriaceae) to evaluate the effect of pollinators and geographical isolation on the bioclimatic niche. These genera possess species characterized by interspecific geographical isolation in different islands and are pollinated by different pollinators. Some species are pollinated by hummingbirds, other by bats, while some are more generalists and are pollinated by pollinators from both functional groups. After describing the bioclimatic niches of plant species, we measured niche overlap for species pairs and we fitted Brownian motion and Ornstein–Uhlenbeck (OU) evolution models with multiple evolutionary regimes to test for an effect of pollination strategy or geographical isolation on bioclimatic niche evolution of these plants. The analysis of niche overlap between plant species, which could not be corrected for phylogenetic relationships, showed that it was significantly influenced by pollination mode and island distribution. By contrast, the best fitting evolutionary model on niche optima and tolerance was always an OU model with a unique selective regime, suggesting that neither pollination strategy nor island isolation had an important effect on bioclimatic niches at a macroevolutionary scale. Instead, we conclude that bioclimatic niches of Antillean Gesneriaceae evolved under phylogenetic conservatism and hypothesize that this macroevolutionary pattern could result from adaptation to temporally variable climates in the Antilles.

## 1. Introduction

Deciphering the mechanisms underlying species evolution is key to understand patterns of biodiversity. Over the years, theoretical and empirical studies have greatly improved our comprehension of the ecological correlates of species formation (e.g. [1,2]). Moreover, the increasing availability of interpolated layers of abiotic variables (e.g. [3]) and of georeferenced specimens under the leadership of the Global Biodiversity Information Facility (GBIF) has allowed investigating patterns and mechanisms of abiotic niche evolution and its relation to species diversification (e.g. [4,5]).

The abiotic niche of species could be important to understand species diversification as it has often been suggested to be a driver of speciation, echoing Simpson’s concept of evolution by niche shifts [6]. For instance, niche shifts were found to be common in the recent radiations of *Pachycladon* [7], *Oenothera* [8], *Anolis* lizards [9] and plethodontid salamanders [10]. By contrast, another commonly reported pattern is the tendency for closely related species to have similar abiotic niches, a macroevolutionary pattern referred to as phylogenetic niche conservatism [11] where ‘species differ less ecologically than might be expected if ecological diversification had occurred in an unconstrained manner’ [12]. According to Wiens [13], the constraints on niche evolution prevent species from adapting to new environmental conditions. In such systems, migration is easier than adaptation [4,14], which favours allopatric speciation and the conservation of niche attributes. This process has been suggested to be the cause of diversification in the legume tribe Indigofereae [15] and in some molluscs of the genus *Corosella* [16], among others.

**Figure 1.**
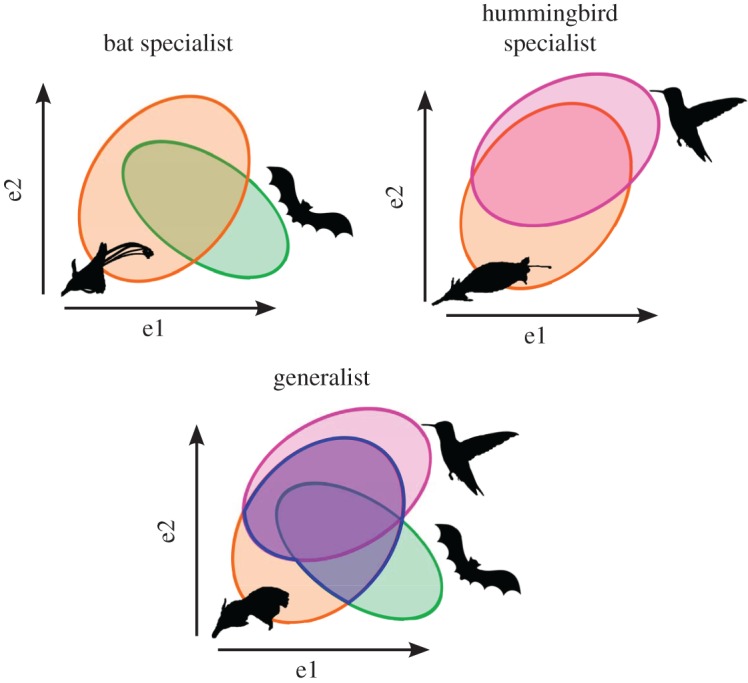
Representation of realized abiotic niches of plants harbouring different pollination strategies. Axes e1 and e2 represent abiotic environmental variables. The three type of plants have the same fundamental abiotic niche (in orange) but their realized niches correspond to the overlap of their own fundamental niche and that of their pollinators (abiotic niche of pollinators in red for hummingbirds and green for bats). Consequently, if pollinator functional groups have different abiotic niches, plants relying on different pollinators should occupy different abiotic realized niches.

Although the study of abiotic niches could help understand the mechanisms of speciation, little attention has been given to factors that could constrain the abiotic niches in this context. For instance, the effect of trophic interactions on abiotic niche evolution has rarely been investigated (but see [17]). The ecological niche of species as defined by Hutchinson [18] is composed of abiotic and biotic factors (i.e. interaction with other species) and their interaction determines the range of the realized niche. Competition, which negatively impacts both interacting species (−/−), trophic interactions (+/−) or mutualism (+/+), are indeed known to play an important role in determining species distributions [19–22] and thus also impact the realized abiotic niches of interacting species. Taking the example of the mutualistic interaction between plants and pollinators, the presence of plants is necessarily limited by the distribution of their pollinators. This constrained distribution represents the realized abiotic niche, which is a fraction of the fundamental niche (figure 1; [23]).

Pollinator species can be grouped into pollinator functional groups that correspond to a group of pollinators exerting similar selective pressures on plant traits (e.g. hummingbirds, bats, bees). Accordingly, two plant species pollinated by different functional pollinator groups should present different realized abiotic niches if their respective pollinator functional groups differ in their abiotic niche, and this even if the plants have the same fundamental abiotic niche. This could be relatively important given that different pollinators can have different ecological niches, notably along elevation gradients [24,25]. Moreover, a generalist species pollinated by several pollinator functional groups could have a wider realized abiotic niche than pollination specialists that are effectively pollinated by only one functional group (figure 1). As an example of such a kind of relationship, in some Caribbean plants pollinated by hummingbirds, the degree of specialization on hummingbirds is correlated with specialization for habitat characteristics such as temperature and rainfall [26]. Additionally, in the Cape flora of South Africa, sympatric sister species differing in pollination mode were adapted to divergent edaphic conditions [27].

Another factor known to affect abiotic niche evolution is the geographical isolation of species; the most illustrative cases of such effects come from the study of oceanic islands. Indeed, niche evolution depends on ecological opportunities (i.e. environmental heterogeneity) and when a species colonizes a new depauperate island, the absence of competition enables it to more easily fill the available environmental space. This way, increased environment heterogeneity on an island should provide greater opportunity for niche diversification. Empirical studies on this subject are numerous and include Darwin finches and the Hawaiian silversword alliance. The intensively studied Antillean *Anolis* lizards represent a model group for the study of island biogeography and niche evolution. In those lizards, the evolution rate of abiotic niches was found to be positively correlated to island size [28]. Geographical isolation is also expected to promote speciation. In this scenario, species diverge by allopatric speciation and if the two new species come into secondary contact, interspecific competition should drive both species niches to diverge (see [29] for a review of effects of islands on speciation and adaptive radiation). Finally, the evolution of ecological niches in distant islands should be independent because of geographical isolation and thus the same niche specialists may have evolved independently in distinct islands (e.g. in *Anolis* different species evolved independently towards the same habitat specialization in different Antillean islands [30]).

In this study, we investigate the potential effect of mutualistic interactions and geographical isolation on the evolution of the abiotic niche of plants from the Gesneriaceae family in the Caribbean region. The plant genera *Gesneria* and *Rhytidophyllum* (Gesneriaceae) together consist of approximately 81 species [31]; they belong to the tribe Gesnerieae that started to diversify approximately 8–11 Ma [32,33]. They present three main pollination strategies (also referred as pollination modes) associated with different pollination syndromes. These pollination strategies correspond to hummingbird specialists, bat specialists and species with a mixed strategy (being pollinated by hummingbirds, bats and sometimes insects) [34]. Unlike plants that rely on a limited number of pollinator functional groups for their reproduction, pollinator species do not rely on a single plant species as their food source. The most recent common plant ancestor was most likely a hummingbird specialist [34], and there have been several subsequent independent evolutions towards bat pollination, either keeping hummingbird pollination (in this case species have a mixed strategy) or losing it (species becoming bat specialists). Some reversals towards the ancestral hummingbird specialist mode were also detected but to a lesser extent [34,35]. The labile nature of pollination strategies gives the opportunity to study biological replicates of pollination transitions, making it easier to test for a link between pollination and abiotic niche evolution. The second interesting aspect of this plant group is its geographical distribution. Indeed, *Gesneria* and *Rhytidophyllum* are endemic to the Antilles, with most of the species being present in only one island of the Greater Antilles (i.e. Cuba, Hispaniola, Jamaica or Puerto Rico [36]) and never on more than two islands. Two species are also present in the Lesser Antilles (*G. ventricosa, R. caribaeum*). Furthermore the Antilles present an important variability of abiotic—particularly bioclimatic—conditions [37] and provide important ecological opportunities for species to fill various abiotic niches. Finally, there is a link between pollination strategies and geographical isolation in the Gesneriaceae family with pollination generalist strategies being far more frequent on islands than on the continent [38], which can be explained by the lower density of pollinators or the absence of certain pollinator functional groups in islands compared to the mainland [38,39].

The presence of geographical isolation (i.e. in five geographical entities: Cuba, Hispaniola, Jamaica, Puerto Rico and the Lesser Antilles), coupled with an important diversity of pollination strategies, allow us to study the effect of both factors on the evolution of realized abiotic niches in this group. Given that, in some plant groups, species pollinated by different pollinators have been shown to occupy different niches and that this was apparently caused by the different niches occupied by the pollinators [26,40], we decided to test whether there could exist an association between abiotic niche evolution and pollination strategies in *Gesneria* and *Rhytidophyllum*. Specifically, we tested (i) whether both pollinator groups (i.e. bats and hummingbirds) had different abiotic niches, which has been suggested previously [25,40] but was never tested for the Antilles; (ii) whether plants with different pollination strategies have divergent abiotic niches and (iii) if pollination generalists harbour wider niches than pollination specialists, and finally (iv) if bioclimatic niches of plants are affected by their geographical distribution. To answer these questions, we first describe bioclimatic niches of pollinators and plants and compare them via niche overlap measurement. Secondly, we fit evolution models on niche components of plants.

## 2. Material and methods

### 2.1. Bioclimatic data and species occurrences

The study area consists of the Lesser and Greater Antilles, which corresponds to the range limit of the studied plant species, even though some pollinators have wider distributions. Altitude and 19 bioclimatic variables were extracted from the Worldclim database [3] with a resolution of 5 arc-min, representing squares of 10 km-long side at the Equator, dividing the study area into 3458 pixels. This resolution was chosen to match the precision of our occurrence data. In a preliminary analysis, we included soil variables (extracted from ISRIC database), but these data did not vary among plant species and were thus not considered further here (results not shown).

Plant occurrence data were obtained from herbarium specimens. Some of them were GPS-referenced during field collection, but the majority were post facto referenced using the GEOLocate Web application [41]. Specimens with uncertain location or with poor coordinate precision (greater than 10 km) were discarded. Secondly, we removed species for which we had less than 5 occurrence records or present in less than five land pixels, which resulted in 49 species. Finally, we did not consider species with no phylogenetic information (see §2.4), leaving 968 unique and reliable occurrences representing 35 species (each species being represented by 6–116 points; electronic supplementary material, table S1).

Hummingbird and nectarivorous bat occurrence data were obtained from GBIF which hosts’ data from, among other sources, MANIS, VertNet and e-bird. Unrealistic data (such as points in the sea) and duplicates were filtered, leaving 299 unique points for bats (eight species, each species being represented by 8–110 points) and 7380 unique points for hummingbirds (14 species, each species having 9–1219 points). Presence points were projected onto a map and none was found to have an aberrant position. Among the 22 pollinators species considered here, three hummingbird species and five bat species have a natural distribution wider than the Caribbean (see electronic supplementary material, table S1), but we considered only presence points within the study area.

### 2.2. Species bioclimatic niche evaluation

We chose to base our study on the environmental space rather than geographical space, as we were interested in adaptation to the environment and not to range distribution. It is also closer to the niche concept of Hutchinson [18]. We thus did not consider species distribution models in this study.

We first described and summarized the environmental conditions available in the study area. A principal component analysis (PCA) based on a correlation matrix, which gives the same weight to all variables in the analysis, was performed on the 3458 land pixels of the raster maps of the Antilles (i.e. the study area). Only the first two principal components were considered in the analysis as they explained most of the bioclimatic variation in the Antilles. Using this two-dimensional ordination of the environmental space, we then characterized the niches of plant and pollinator species. The presence points of species were projected on the first two axes of the environmental space, keeping only one presence record per pixel per species to account for sampling bias. This is important as plant collectors often return to the same localities. Niche identity and breadth were estimated for each environmental axis. The niche identity consists of the mean score value of all the points for each species along the axis, while the niche breadth was measured as the standard deviation of these scores (which is not influenced by the number of individuals per species). With this procedure describing the environmental space over the Antilles (even in geographical space where no species is present), environmental axes do not necessarily represent the most important variables for discriminating species niches. Other methods such the as outlying mean index [42] enable selecting components that correlates with variables explaining most of the differences between species. However, using this type of approach would not have allowed addressing the same questions. Indeed, analysing pollinators and plants together using the outlying mean index would have given axes important for differentiating pollinators from plants while we wanted to focus on plant niches. And the other option of analysing plants and pollinators separately would have made overlap estimation impossible (as axes would have been different for plants and pollinators). Moreover, the method we chose has been shown to perform quite well in comparison with other methods for estimating niche overlap [43].

### 2.3. Niche overlap

We tested whether polllinator functional groups had divergent bioclimatic niches and whether plant species with different pollination strategies also had different bioclimatic niches. Both hypotheses were tested the same way, via niche overlap measurement. Niche overlap for each species pair was measured according to the method developed by Broennimann *et al.* [43], using the D index of Schoener as described by Warren *et al.* [44]. We measured niche overlap for plant species pairs and pollinator species pairs and then tested if different variables could explain the D value using linear models. For plant comparisons, four linear models were built including as explanatory variable (i) the fact of sharing the same pollination strategy, (ii) the fact of occurring on the same island, (iii) both of these variables, or (iv) both variables and their interaction. The same was done for pollinator comparisons replacing ‘the fact of sharing the same pollination strategy’ by ‘the fact of belonging to the same functional group’. The best model was selected with AIC value and we report *R*^2^ and *p*-value of the best fitting model. As D is a distance, this analysis was not phylogenetically corrected.

To ensure that bioclimatic niches of plants were included in the niche of their pollinator functional groups, we compared the range of the niches (values between the most extreme points) of plant and pollinator species for the first two principal components.

### 2.4. Bioclimatic niche evolution among plants

**Figure 2.**
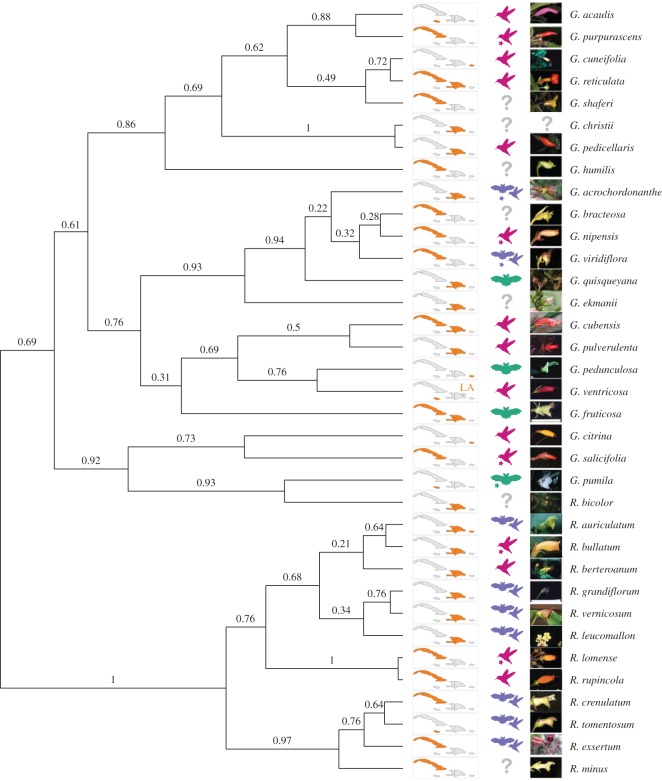
Phylogeny of *Gesneria* and *Rhytidophyllum*. Numbers over the branches represent posterior probabilities. To the right of the phylogeny, the first column corresponds to the geographical distribution of each species (orange: presence, grey: absence, LA: present in the Lesser Antilles). The second column represents pollination mode (question mark: unknown, green: bat specialist, purple: mixed strategy bat–hummingbird, pink: hummingbird specialist; asterisks indicate species for which the pollination mode has been inferred from morphological data; see the electronic supplementary material for details). The third column represent floral phenotypes (photos from John Clark and Simon Joly).

To test the hypothesis that the bioclimatic niche of plants is constrained by pollination strategy and geographical isolation between islands on a macroevolutionary timescale, we fitted evolutionary models on a phylogeny reconstructed in a previous study [35]. This phylogeny is a species tree built with five single copy nuclear genes (*CYCLOIDEA, GAPDH, CHI, F3H* and *UF3GT*) with the Bayesian *beast algorithm, including 45 *Gesneria–Rhytidophyllum* species and three outgroup species (figure 2). As presence points and pollination data were available only for 35 species, we discarded 10 species from the phylogeny for subsequent analysis. The study of niche evolution among plants was done by fitting several evolution models on the data and comparing them with AIC. This involved two steps. To fit evolution models depending on a specific factor (e.g. the pollination mode) on a phylogenetic tree, each branch must first be attributed a state of the trait (e.g. hummingbird specialist or generalist) that will indicate under which selective regime the niche is evolving. Once this is done, the second step consists in fitting the evolutionary models for the observed niche values. As the trait states of the branches of the phylogeny are not known, we reconstructed ancestral states for both traits with stochastic character simulation (i.e. pollination strategy and island distribution; see below). We then fitted evolution models to niche identity and breadth as described below. To account both for niche and phylogenetic uncertainties, the measures were performed over 1000 resampled niche values and over 1000 trees randomly sampled from the posterior distribution of the *beast analysis. The 1000 resampled niche values were obtained by sampling half of the presence points (50% jackknife).

#### 2.4.1. Stochastic character simulation

##### 2.4.1.1. Geographical distribution

We considered four states for the geographical trait: Cuba, Hispaniola, Puerto Rico and Jamaica. As only one species from our phylogenetic dataset is present in the Lesser Antilles, and because this species also occurs in Jamaica (*G. ventricosa*), we chose to discard the Lesser Antilles from our analysis. As states must be exclusive for fitting the evolution models, we decided to sample randomly one of the islands for species that are present in two islands (i.e. for *G. cubensis, G. fruticosa, G. reticulata* and *R. auriculatum*), for each of the 1000 iterations. The stochastic mapping was performed with a symmetric transition rate between states.

##### 2.4.1.2. Pollination mode

In this study, we performed the analyses at the level of functional groups for the pollinators. Therefore, species observed to be pollinated by different species of hummingbirds were assumed to share the same pollination mode. Although this approach removes information, it seems appropriate in the present context because the observation of clear pollination syndrome phenomena [35,45] suggests that different species from the same pollinator group can exert similar selection pressure on plants. Among the 35 plant species, 20 had a pollination mode confirmed with field observations [34,38,45,46]. The 15 others had a pollination mode inferred from their floral shape [35]. We thus ran analyses both for the 20 species set and the 35 species set. Details on pollination mode determination is given in electronic supplementary material, table S2. For the stochastic mapping performed with the inferred syndromes, we incorporated uncertainty for the prior probabilities of the inferred pollination syndromes. Because specialist syndromes can be determined with greater accuracy [45], a prior probability of $\frac{2}{3}$ was given to the inferred specialist mode, whereas a prior probability of $\frac{1}{2}$ was given for a generalist inferred mode. In each case, the other modes were given equal probabilities. For instance, if a hummingbird specialist mode is inferred, the hummingbird, bat and generalist modes were given a prior probability of $\frac{2}{3}$, $\frac{1}{6}$ and $\frac{1}{6}$, respectively. By contrast, pollination syndromes obtained from field observations were given a prior probability of 1. Finally, a prior probability of $\frac{1}{3}$ was assigned for species with no shape data or for which shape did not enable a clear inference. Note that we considered *G. humilis* as having an unknown pollination strategy in this study, while a recent paper described it to be pollinated by insects [38]. The stochastic mapping was performed with an ‘all rate different’ model for transition rate between states. Because the results were similar between the two sets of analyses, we only show the results obtained with the 35 species set.

For both geographical distribution and pollination mode, ancestral traits were reconstructed with stochastic mapping [47] with the function *make.simmap* of the package phytools v. 0.3-72 [48].

#### 2.4.2. Evolution models fitting

Six evolution models were fitted on the data: (i) a Brownian motion model with one rate of niche variation for the whole phylogeny (BM1); (ii) a Brownian motion model in which each pollination mode has its own rate of niche variation (BM3); (iii) a Brownian motion model in which each island has its own rate of niche variation (BM4); (iv) an Ornstein–Uhlenbeck (OU) model with one selective regime over the phylogenetic tree (OU1); (v) an OU model with a different selective optimum for each pollination mode (OU3); and (vi) an OU model with a different selective optimum for each island (OU4). The OU model differs from the BM model by having an optimal trait value (for each regime) and a parameter that determines the strength of selection to bring the variation closer to the optimum. When the selection parameter equals 0, the OU model becomes a BM model [49]. The biological interpretation of the fit of evolution models can vary depending on the specific situation, but common interpretation is that Brownian motion can result from drift or fluctuating selection [50], whereas OU models are often interpreted as evidence of either stabilizing or directional selection, depending on the ancestral value. Another interpretation of OU models is that the evolution of niches is less affected by the phylogeny, especially for distantly related species [50].

The models were fitted on the niche identity and on niche breadth for environmental axes separately (univariate model) and simultaneously (multivariate model). Model fitting was done with the functions *mvBM* and *mvOU* of the package mvMORPH v. 1.0.3 [51]. Models were compared using the AICc weight according to the formula described by Burnham & Anderson [52] after having computed AICc following the formula

2.1 $$\mathrm{AICc}=\mathrm{AIC}+\frac{2k(k+1)}{n-k-1},$$

where *k* is the number of estimated parameters, *n* is the number of species × the number of variables analysed (1 for univariate analysis or 2 for multivariate analysis).

## 3. Results

### 3.1. Species bioclimatic niche evaluation

**Figure 3.**
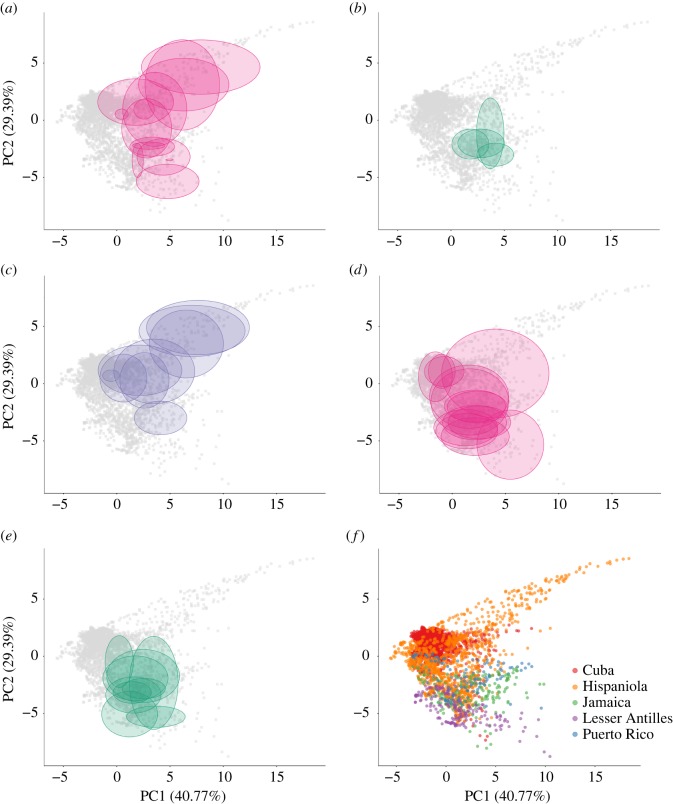
Bioclimatic niches of plants and pollinators. (*a*) Hummingbird specialists, (*b*) bat specialists, (*c*) generalists, (*d*) hummingbirds and (*e*) bats. (*f*) The projection of the pixels of the major islands and Lesser Antilles on the PCA. The grey squares in graphs (*a*–*e*) represent available bioclimatic condition over the Antilles (pixels of all the regions).

The first two principal axes of the PCA represented together 70.16% of the environmental variation present on the islands and the species are widely distributed over the environmental space (figure 3, details on niche identity and breadth over PC1 and PC2 are given in electronic supplementary material, table S3). Although no bioclimatic variable is clearly linked to only one principal axis, the first axis corresponds to a gradient from high-temperature and low-rainfall areas to areas with lower temperatures and higher rainfall, while the second axis is more related to seasonality (see electronic supplementary material, table S4 and figure S1). Although the third and fourth principal components represented a moderate portion of the environmental variance, we focused our analysis on the first two components for practical reasons. Indeed, the estimation of niche overlap (D) using Broennimann *et al.*’s method [43] is only implemented for two niche dimensions and analysing more than two variables for the evolution models fitting would have been statistically difficult for the multivariate model due to the increased number of parameters to estimate. However, the third and fourth axes did not permit to discriminate species as well as the first and second axes and thus were considered of less importance for the present study, especially since the PCA was conducted without considering species’ presence. Indeed, the variance explained for the environmental space for the third and fourth axes (i.e. 13.81% and 6.98%) does not necessarily represent an important aspect for species niche distinction (this is illustrated in electronic supplementary material, figure S2).

### 3.2. Inclusion of bioclimatic niches of plants in bioclimatic niches of their pollinators and niche overlap

#### 3.2.1. Inclusion of niches of plants in niches of pollinators

**Figure 4.**
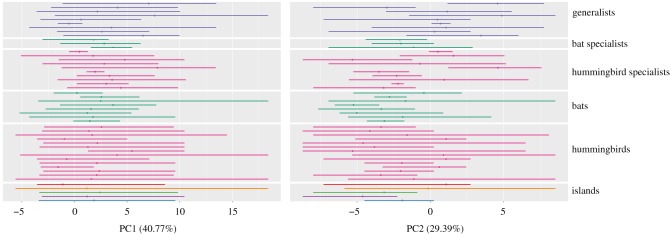
Niche identity (point) and range (line) of plant (hummingbird specialists, bat specialists and generalists) and pollinator (hummingbirds, bats) species over the first and second principal components. The mean and range of each major island and the Lesser Antilles are also shown (figure 3 for islands’ colour code).

The pollinator and plant species occurred over a large part of the available environment (figure 3). The whole distribution of plants over the first and second principal components was included in the distribution of their pollinator functional groups (figure 4). Consequently, we consider that the realized bioclimatic niches of plants are included in the bioclimatic niche of their respective pollinator functional groups.

#### 3.2.2. Plants overlap

The best model explaining niche overlap (D) between plants included the pollination strategy, geographical information (island) and the interaction between both variables (table 1). This suggests that the similarity between the bioclimatic niches of plants depends on island distribution and on pollination strategy of species, which is in accordance with our expectations. Note that this model explains 17.33% of the variance, suggesting that other unconsidered aspects greatly influence bioclimatic niches of plants.

**Table 1.** AIC, *R*^2^ and regression coefficients of linear models for the niche overlap of plant species. ‘pollination’ refers to the fact of sharing the same pollination mode and ‘island’ refers to the fact of occurring on the same island. Significance of each variable is represented by ‘*’ and ‘.’ (*p*-values: ***<0.001<**<0.01<*<0.05<.<0.1).

model	AIC	pollination	island	interaction	intercept	*R*^2^
D ∼ 1	−66.33	—	—	—	—	—
D ∼ pollination	−64.47	0.01	—	—	0.21***	−0.00
D ∼ island	−100.77	—	0.16***	—	0.15***	0.14
D ∼ pollination + island	−99.95	0.03	0.17***	—	0.14***	0.14
D ∼ pollination * island	−107.32	−0.03	0.11***	0.17***	0.16***	0.17

#### 3.2.3. Pollinators overlap

The best model explaining niche overlap (D) between pollinators included the pollinator functional group and the geographical information (island), but not the interaction between both variables (table 2). However, the simpler model with only the island as explanatory variable cannot be completely rejected as it is within 2 AIC units from the best model. These results suggest that although pollinators from the same functional group tend to be more similar, this is not a very good predictor of niche similarity. By contrast, the fact of being on the same island generally results in greater niche overlap for pollinators. Again, as these models explain less than 5% of the variance in niche overlap, niche identity of pollinators is certainly also influenced by pollinator species in addition to their functional group.

**Table 2.** AIC, *R*^2^ and regression coefficients of linear models for the niche overlap of pollinator species. ‘group’ refers to the fact of belonging to the same pollinator functional group and ‘island’ refers to the fact of occurring on the same island. Significance of each variable is represented by ‘*’ and ‘.’ (*p*-values: ***<0.001<**< 0.01<*<0.05<.<0.1).

model	AIC	group	island	interaction	intercept	*R*^2^
D ∼ 1	−109.87	—	—	—	—	—
D ∼ group	−110.34	0.04	—	—	0.31	0.01
D ∼ island	−117.71	—	0.08**	—	0.29***	0.04
D ∼ group + island	−119.46	0.05 .	0.08***	—	0.27***	0.05
D ∼ group * island	−118.47	0.02	0.06	0.05	0.28***	0.05

### 3.3. Bioclimatic niche evolution among plants

The species phylogeny we used is concordant with previous studies [34] and is described in more detail elsewhere [35]. Some branch support values were quite high although some relationships among closely related species were ambiguous (figure 2), indicating the importance of considering phylogenetic uncertainty in our analyses.

We present here only the results for the analysis performed on the 35 species set (i.e. with both species with known and inferred pollination mode) but the analysis on the 20 species set (with only species with a known pollination mode) was concordant. The OU1 model was the best fitting model for all analyses performed, with mean AICc weights above 0.69 (table 3). Moreover, all BM models received AICc weights of 0. This supports the presence constraints that keep the species bioclimatic niche optima and breadth more similar to each other than what is expected under a Brownian model of evolution. The OU models with three and four regimes always had a poorer fit than the models with one regime, suggesting that plants with different functional pollinators and on different islands do not have distinct niche identity or breadth. However, the weight for these models was not null and as such we cannot completely reject this hypothesis, somewhat supporting the weak relationships obtained with the analyses of niche overlap.

**Table 3.** Mean AICc weights of evolution models fitted on niche identity and breadth. Numbers in brackets represent 95% CI.

variable	BM1	BM3	BM4	OU1	OU3	OU4
niche identity (PC1)	0 [0.00–0.00]	0 [0.00–0.00]	0 [0.00–0.00]	0.70 [0.07–0.91]	0.18 [0.03–0.80]	0.11 [0.01–0.68]
niche identity (PC2)	0 [0.00–0.00]	0 [0.00–0.00]	0 [0.00–0.00]	0.70 [0.10–0.90]	0.19 [0.03–0.82]	0.11 [0.01–0.72]
niche identity (PC1 and PC2)	0 [0.00–0.00]	0 [0.00–0.00]	0 [0.00–0.00]	0.73 [0.09–0.97]	0.16 [0.01–0.78]	0.11 [0.00–0.75]
niche breadth (PC1)	0 [0.00–0.00]	0 [0.00–0.00]	0 [0.00–0.00]	0.70 [0.08–0.91]	0.18 [0.02–0.70]	0.12 [0.01–0.70]
niche breadth (PC2)	0 [0.00–0.00]	0 [0.00–0.00]	0 [0.00–0.00]	0.69 [0.11–0.90]	0.18 [0.02–0.79]	0.12 [0.01–0.74]
niche breadth (PC1 and PC2)	0 [0.00–0.00]	0 [0.00–0.00]	0 [0.00–0.00]	0.73 [0.07–0.97]	0.14 [0.01–0.74]	0.12 [0.00–0.85]
niche identity and breadth (PC1)	0 [0.00–0.00]	0 [0.00–0.00]	0 [0.00–0.00]	0.72 [0.04–0.97]	0.16 [0.01–0.86]	0.12 [0.00–0.84]
niche identity and breadth (PC2)	0 [0.00–0.00]	0 [0.00–0.00]	0 [0.00–0.00]	0.72 [0.04–0.97]	0.15 [0.01–0.89]	0.13 [0.00–0.87]

## 4. Discussion

Our study aimed at documenting the evolution of bioclimatic niches in relation to pollination mode and geographical isolation in a group of insular plants. Biotic interactions and geographical isolation are known to influence abiotic niche evolution in some groups [26,40]. However, addressing both traits concomitantly has rarely been done, even though investigating the interactions between these is important to understand what drives the colonization of new niches in plant evolution.

### 4.1. Evolution of bioclimatic niches of plants was not strongly influenced by pollination mode and island distribution

When analysing niche overlap, we observed that pollination modes did not strongly influence niche similarity for plants. Interestingly, the main effect of the pollination mode on niche overlap (D value) was via its interaction with the island variable (i.e. the fact of occurring or not on the same island), which is discussed below. A weak impact of pollination mode on niche components can similarly be reached with the results from the evolution models, as models with one selective regime obtained a better fit than models with one regime per pollination syndrome for both niche identity and tolerance. These results are probably caused by the fact that the bioclimatic niches of hummingbirds do not differ importantly from those of bats. Indeed, contrary to our initial expectation, hummingbirds and bats were found to have similar abiotic niches. Therefore, it is not surprising that we did not observe different niche identity or breadth between plants with different pollination modes. This result is comparable to that of Serrano-Serrano *et al.* [53] who worked on other Gesneriaceae tribes widely distributed on the South American continent and showed that bioclimatic niche evolved independently from floral traits.

Geographical isolation, which was included in the model by using the information on the island of provenance of species, was found to affect the niche overlap between plants. Plant-realized bioclimatic niches were found to be more similar when plants occur on the same island (mean niche overlap [D] of 0.31 for species on the same island and 0.15 for species on different islands). This may be partly explained by the fact that the available environments are globally more similar within islands and that pollinators are not bioclimatically restricted within islands (figure 3). However, the evolution models allowing a specific selective regime per island (BM4 and OU4) received less support than OU models with one selective regime, suggesting that island identity alone did not significantly constrain niche components on evolutionary timescales.

Interestingly, we found that the interaction between pollination mode and island distribution could affect plant niches. Indeed, plants with a generalist pollination strategy have far more similar bioclimatic niches when they are on the same island than when they occur on different islands (electronic supplementary material, figure S3). On the contrary, the overlap between the bioclimatic niches of hummingbird specialists is little affected by the geographical provenance of species (see electronic supplementary material, figure S3). However, one limit of current evolution models is that none of them include the possibility for species to be evolving simultaneously under two (or more) selective regimes. Such a limit forced us to consider species to be present in only one island even when they were present in two, potentially reducing the power of our models.

One potential explanation for the fact that the pollination mode was not significant in this study could be that these categories do not represent the true selective regimes. This is possible given that hummingbird specialists are generally pollinated by different hummingbirds on different islands, because these species are usually island endemics, but generally pollinated by the same hummingbird on a given island [38]. This could result in an interaction effect between pollination strategy and geographical distribution that could remove power from the phylogenetic analyses as interaction evolution models do not exist yet.

Finally, our hypothesis that plants with a generalist pollination strategy could occupy wider niches than plants with a specialist strategy was rejected as the fit of evolution models on niche tolerance favoured the model OU1 with one constant selective optimum over plant groups. In the light of the results of niche overlap between pollinator species of different functional groups (Results §iii), it seems evident that if pollinator functional groups do not have divergent bioclimatic niches, generalist plants cannot occupy wider niches than specialists. This result is interesting *per se* as it suggests that becoming generalist does not represent an advantage for plant species in terms of niche colonization (which could decrease the competition pressure between species).

### 4.2. Evolutionary constraints on niche components

The model best explaining niche evolution was the OU model with one selective regime (OU1, table 3). The selection of the OU1 model, with one selective regime across the whole tree, is concordant with a hypothesis of niche conservatism [11]. Indeed, it means that species are more similar to each other than what would be expected under a BM model in which traits evolve without constraints along the phylogeny [12]. A conclusion of phylogenetic niche conservatism has to be taken cautiously, however, because other explanations could explain such a pattern. For instance, Boucher *et al.* [54] showed that, in a bounded landscape, boundaries restrain the range of available conditions, which artificially result in the niche evolution having a better fit to an OU model than a BM model when the bounds are reached, and this even if species evolve independently from the environment. Our results show that while the plant group is present in a broad range of conditions, few species reach the boundaries of niche space (figures 3 and 4), suggesting that this is unlikely to affect our results.

OU1 was the best fitting model when analysing niche identity as well as niche breadth. This is generally interpreted as a constrained evolution towards a global optimum. However, Antillean islands frequently experience important climatic variability both in temporal and spatial scales [55] and were also subject to high climate variability during the Pleistocene [56]. Such temporal variability associated with limited migration opportunities within and among islands could result in the homogenization of niches, both in identity and breadth, resulting in a pattern of phylogenetic niche conservatism. Indeed, several experimental evolution studies have shown that selection in temporally varying environments lead to wider niches (reviewed in [57]). And in Eriogonoideae plant species, broader climatic tolerances in perennial species were associated with regions characterized by greater environmental instability [58]. In the present group, temporal climatic variation could similarly have affected the populations of plants and pollinators and could have resulted in fluctuating selection on the optimal niche for the species, creating a pattern of bioclimatic niche stasis.

### 4.3. Conclusion

In line with our initial hypothesis based on obligate mutualist interactions, we found that the bioclimatic niches of plants were always included within those of their functional pollinators. By contrast, although we observed that plants with the same functional pollinators or on the same island tended to be more similar (i.e. they showed increased overlap D), these factors were not found to significantly affect the evolution of the plant bioclimatic niches through time in this group. This might in part be explained by the fact that the different functional pollinators were not found to differ strongly in their bioclimatic niches. Instead, bioclimatic niches of plants seem to have evolved under phylogenetic niche conservatism, a pattern that could result from the homogenization of niches caused by past climatic fluctuations in the Antilles.

## Supplementary Material

Script and raw data;supplementary informations

## Data accessibility

Bioclimatic and altitude data are available on http://www.worldclim.org/. R scripts and raw data are available as the electronic supplementary material. The supplementary file bioclimatic_niches_script.html contains a description of the analyses performed in this study as well as the R scripts. All the data necessary to run the scripts are in the compressed file supplementary_data.zip.
